# Indolizidines and quinolizidines: natural products and beyond

**DOI:** 10.1186/1860-5397-3-27

**Published:** 2007-09-26

**Authors:** Joseph P Michael

**Affiliations:** 1 Molecular Sciences Institute, School of Chemistry, University of the Witwatersrand, PO Wits 2050, South Africa

Alkaloids occur in such astonishing profusion in nature that one tends to forget that they are assembled from a relatively small number of structural motifs. Among the motifs that are most frequently encountered are bicyclic systems containing bridgehead nitrogen, especially 1-azabicyclo[4.3.0]nonanes and 1-azabicyclo[4.4.0]decanes or their unsaturated analogues – the indolizidines and quinolizidines to which this Thematic Series is devoted. These two azabicyclic systems may occur in the natural products either in isolation (the so-called 'simple izidine' alkaloids) or, more commonly, embedded within fused polycyclic arrays. Just how widespread they are was pointed out over two decades ago in a review in which it was estimated that between 25% and 30% of all alkaloids possess structures incorporating one or other of these motifs. [1] As might be expected of systems that are so pervasive, their natural sources are extremely diverse: they occur in organisms as widely different as bacteria, fungi, higher plants, invertebrates and vertebrates; and both terrestrial and marine sources are represented. For example, two of the best-known and most widely investigated groups of 'simple izidine' alkaloids are the plant-derived polyhydroxylated indolizidines that function as potent glycosidase inhibitors, [2–4] and the alkylindolizidines and analogues sequestered from dietary sources in the skins of amphibians. [5–6] It is thus hardly surprising that both the structural elucidation and the total synthesis of these and related alkaloids continue to attract the attention of eminent chemists, as borne out by the seemingly inexhaustible flow of publications in prominent journals. [7] Several general reviews on these alkaloids have also appeared in recent years. [8–11]

The interest in indolizidines and quinolizidines, although inspired by alkaloids, nowadays extends far beyond natural product chemistry. Considerable effort is being invested in the development of innovative methods for preparing the parent bicyclic systems and, more especially, for the stereocontrolled attachment of substituents. Studies on the biological activity of compounds containing azabicyclic building blocks (for example, rigid bicyclic peptidomimetics) are gaining momentum. Structural, spectroscopic and computational studies on both natural and synthetic indolizidines and quinolizidines are also reported regularly. In this Thematic Series, there are representative articles covering several of these aspects. A number of authors have contributed reviews in which their own contributions to the development of indolizidine and quinolizidine chemistry are highlighted. There are articles on the total synthesis of relevant natural products, as well as articles describing novel methodological approaches to the systems of interest. That what may appear to be a marginal, passé or recondite outpost of chemistry still attracts a healthy measure of international attention bears testimony to the durability of a topic that is sure to retain its fascination for the foreseeable future.

Jo Michael

Guest Editor
